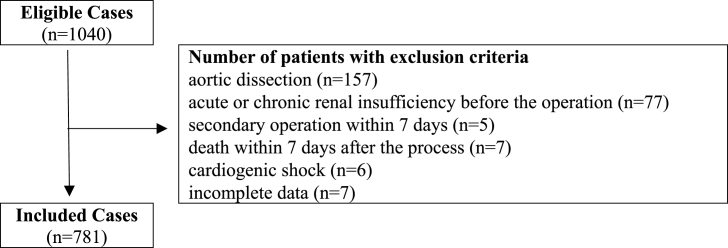# Gender differences and risk factors for acute kidney injury following cardiac surgery: A single center retrospective cohort study

**DOI:** 10.1016/j.heliyon.2023.e22177

**Published:** 2023-11-14

**Authors:** Yichuan Wang, Xuliang Huang, Shanshan Xia, Qingqing Huang, Jue Wang, Maochao Ding, Yunchang Mo, Jianping Yang

**Affiliations:** a Department of Anesthesiology, The First Affiliated Hospital of Soochow University, China; b Department of Anesthesiology, The First Affiliated Hospital of Wenzhou Medical University, China; c Department of Cardiac Surgery, The First Affiliated Hospital of Wenzhou Medical University, China; d Department of Human Anatomy, Wenzhou Medical University, China

**Keywords:** Acute kidney injury, Gender, Cardiac surgery, Cardiopulmonary bypass

## Abstract

**Background:**

We studied AKI incidence and prognosis in cardiac surgery patients under and over 60 years old.

**Methods:**

We studied AKI in patients who underwent cardiac surgery at the First Affiliated Hospital of Wenzhou Medical University between Jan 2020 and Dec 2021, using improved global prognostic criteria for diagnosis.

**Results:**

After analyzing 781 patients (402 males, 379 females), AKI incidence after surgery was 30.22 %. Adjusting for propensity scores revealed no significant difference in AKI incidence between young males (24.1 %) and females (19.3 %). However, young females had higher AKI stages. Among older patients, AKI incidence was comparable between males (43.4 %) and females (42.2 %), but females had longer intubation times. Independent risk factors for AKI included age, male gender, and BMI, while intraoperative hemoglobin level was protective.

**Conclusions:**

No gender gap in AKI frequency for <60 years old and ≥60 years old post-cardiac surgery, yet women display increased AKI severity and extended intubation duration.

## 1 Introduction

Acute kidney injury (AKI) is a rapid decline in kidney function caused by various factors. It is characterized by a decrease in glomerular filtration rate, leading to water and sodium retention, electrolyte imbalances, and systemic symptoms affecting multiple body systems. Cardiac surgery with cardiopulmonary bypass is a common cause of AKI. Previous studies have suggested that the harm caused by CPB to the human body includes systemic inflammatory reactions, hemodilution, and nonpulsatile perfusion [1]. Depending on the evaluation criteria used, the probability of AKI after cardiac surgery ranges from 20 % to 40 % [2]. Despite years of research, acute kidney injury following cardiac surgery remains a significant problem. Identifying the risk factors for kidney injury is crucial in order to implement targeted strategies.

Gender differences have been widely studied in relation to their impact on clinical outcomes. This field of research has explored various conditions such as coronary atherosclerotic heart disease, acute ischemic cerebrovascular accident, autoimmune diseases, and more [[3], [4], [5], [6]]. A retrospective cohort study of 390,382 non-cardiac surgery patients with acute kidney injury found that the incidence of AKI after surgery was lower in women under the age of 50, and that the incidence of AKI gradually increased with age [7]. So far, there is still controversy over the gender differences in the occurrence and prognosis of AKI after cardiac surgery, and some studies suggest that males have a higher incidence of AKI [[8], [9], [10], [11]]Some studies did not observe this difference [12].

Young women with higher levels of sex hormones may have a protective effect on the kidneys. However, regardless of gender, after the age of 60, the levels of sex hormones in the body decrease significantly [13,14]. Our study set the age of 60 as the boundary and divided all cases into younger and older groups to observe the differences in the incidence and prognosis of postoperative acute kidney injury (AKI) between the two groups. Due to significant differences in surgical type, smoking, alcohol consumption, and baseline kidney function between men and women, we used propensity score matching analysis for the preoperative and intraoperative basic characteristics to eliminate confounding factors and accurately reflect gender differences in AKI. We conducted a risk factor analysis for all study populations, assuming that gender and age are risk factors for postoperative AKI in cardiac patients. We hypothesized that in the population undergoing cardiopulmonary bypass heart surgery, the incidence of acute kidney injury (AKI) is lower in young females compared to young males, and that male gender is an independent risk factor for AKI after cardiac surgery.

## 2 Methods

### 2.1 Design, settings, and participant selection

All procedures in this study were in accordance with the Helsinki Declaration (revised in 2013), and the Ethics Committee at the First Affiliated Hospital of Wenzhou Medical University approved the study. As the researchers did not carry out any additional interventions, the requirement for an informed consent form was waived. The ethics approval number is KY2023-R214. The full name of the ethics committee is Ethics Committee in Clinical Research of the First Affiliated Hospital of Wenzhou Medical University.

This is a retrospective cohort study conducted at the First Affiliated Hospital of Wenzhou Medical University, focusing on adult patients who underwent cardiac surgery between January 2020 and December 2021. All patient variables, including medical history, laboratory tests, echocardiography, and intraoperative blood gas analysis, were retrieved from the electronic medical records database. The cardiac surgery team at the hospital performed all the operations, and the patients were subsequently transferred to the cardiac intensive care unit (CCU) for further monitoring. All patients used the same cardiopulmonary bypass system. The types of surgeries performed included heart valve disease (HVD), congenital heart disease (CHD), coronary artery bypass grafting (CABG), and combined heart surgery. Combined heart surgeries included ascending aorta replacement (AAR), AAR + HVD, AAR + CABG, HVD + CHD, HVD + CABG.

Inclusive criteria for this study included: (1) age equal to or greater than 18 years, and (2) the use of cardiopulmonary bypass under mild hypothermia during the operation. Exclusion criteria consisted of: (1) severe arrhythmia or hypotension prior to the procedure (blood pressure at hospitalization <90/50 mmHg, where 1 mmHg = 0.133 kPa), (2) preoperative renal insufficiency (GFR <60 mL/min/1.73m2) or cardiogenic shock, (3) large vessel surgery requiring deep hypothermic circulatory arrest, and (4) second operation or death within 7 days after the operation. The patients who participated in this study were divided into two groups, an old group and a young group, based on age, with the cutoff being 60 years.

### 2.2 Data collection

The research data for this study was sourced from the electronic medical record database of the First Affiliated Hospital of Wenzhou Medical University. The statistics and perioperative data analyzed in this study include: age, gender, height, weight, preoperative echocardiography and laboratory examination, previous medical histories such as myocardial infarction, CPB time, arterial blood gas examination during cardiopulmonary bypass, and postoperative laboratory examination. The preoperative glomerular filtration rate (GFR) was calculated using the Modification of Diet in Renal Disease equation (GFR = 186.3 × serum creatinine level (mg/dL)^−1.154^ × age^−0.203^ × 0.742 (if female)) [15]. Patients with a calculated GFR of <60 mL/min/1.73 m^2^ were excluded.

### 2.3 Diagnosis of AKI

The diagnosis of AKI was made using the improved global prognostic criteria (KDIGO) by comparing the postoperative serum creatinine level with the preoperative serum creatinine level [16]. Patients were classified into stages 1–3 of AKI. Stage 1 was defined as an increase in serum creatinine by more than 26.5 μmol/L within 2 days after cardiac surgery, or an increase in serum creatinine to more than 1.5 times the preoperative level within 7 days after the procedure. Stage 2 was defined as an increase in serum creatinine to 2.0–2.9 times the baseline level. Stage 3 was defined as an increase in serum creatinine to 3 times baseline or ≥4.0 mg/dL (≥353.6 μmoL/l) increase, or initiation of RRT. The KDIGO urine volume standard for AKI was not utilized in this study.

### 2.4 Surgical techniques

The management of cardiopulmonary bypass (CPB) involved administering intravenous heparin to achieve an activated clotting time greater than 480 s. The types of surgeries performed included open heart surgery and video-assisted thoracoscopic heart surgery. High potassium 4:1 blood cardioplegia or DelNido cardioplegia and standard CPB tubes were used. The targeted mean perfusion pressure ranged between 50 and 80 mmHg, and pump flow rates of 2.0–2.5 L/min/m2 were maintained. Concentrated packed red blood cells were transfused during CPB if hemoglobin levels fell below 7 g/dL.

### 2.5 Statistical analysis

Categorical variables were presented as frequencies with percentages and compared using either the Chi-square test or Fisher's exact test, as appropriate. Continuous variables with a normal distribution were expressed as means ± standard deviation (SD) and compared using the *t*-test. Continuous variables without a normal distribution were expressed as medians with interquartile ranges (IQR) corresponding to the 25th and 75th percentiles and compared using the Wilcoxon ranked sum test.

Differences in patient characteristics between males and females in the two groups were adjusted by propensity score matching (PSM). The researchers conducted PSM analysis on the data using SPSS 25.0 system. The propensity score was based on potential confounders, patient-related characteristics and intraoperative related characteristics. PSM was conducted in a 1:1 ratio with a matching tolerance value of 0.1, prioritizing exact matches. A total of 83 pairs of data were matched for both the young and elderly groups. After matching, baseline comparisons of the data showed p-values greater than 0.05, indicating comparability of the matched data. Finally, we estimated the relative risk (RR) and its 95 % confidence interval (CI) for acute kidney injury (AKI) among males and females.

Furthermore, we constructed a multivariable logistic regression model for all patients. All reported P values were two-tailed, and statistical significance was defined as P < 0.05. Data analysis was performed using SPSS version 25.0 (IBM SPSS Software for Predictive Analytics; SPSS, Chicago, IL, USA).

## 3 Result

### 3.1 General information

Between January 1, 2020, and December 31, 2021, a total of 1040 adult patients underwent cardiac surgery with cardiopulmonary bypass (CPB) at the First Affiliated Hospital of Wenzhou Medical University. Among these patients, 781 were included in the study (see appendix) and had a mean age of 57.92 ± 12.99 years, with a range of 18–85 years. Of these patients, 402 (51.47 %) were male. Among the 781 patients, 236 were diagnosed with acute kidney injury (AKI), resulting in an incidence of 30.22 % after surgery. The incidence of AKI was higher in the older group, with 40.7 % (161/396) of patients over the age of 60 developing AKI, compared to 19.5 % (75/385) of patients in the younger group.

### 3.2 Comparison of gender differences in young and old groups before propensity scroe mathing

Based on the results, male patients in both groups had higher levels of weight, height, and preoperative hemoglobin. Male patients also showed a higher incidence of smoking, alcohol consumption, and myocardial infarction. Young males had a higher BMI compared to females, and young males were more likely to have a history of hypertension, while old females were more likely to have a history of diabetes.

In terms of preoperative factors, female patients in both groups had lower sCr levels and GFR compared to male patients. Additionally, old males had lower preoperative LVEF than females.

There was also a significant difference in the type of surgery between male and female patients in both groups (Table 1).

**Table 1:** Demography and clinical characteristics before Propensity Scroe Mathing.

Characteristics	Young group (age<60)				Old group (age≥60)		
	Male	Female	*P* value	Male		Female	*P* value
	(n = 183）	(n = 202）		(n = 219)		(n = 177）	
**Patient-related characteristics**							
Age（years）^b^	50（45,55）	50（43,55）	0.608	68（64,72）		67（64,71）	0.608
Weight（kg）^a^	67.2 ± 10.3	55.3 ± 9.0	0.000	62.8 ± 8.6		54.5 ± 9.5	0.000
Height（cm）^a^	169.4 ± 6.1	157.0 ± 6.1	0.000	165.6 ± 12.5		155.4 ± 5.8	0.000
BMI (kg/m^2^)^a^	23.4 ± 3.2	22.4 ± 3.2	0.004	22.7 ± 2.8		22.5 ± 3.5	0.667
Smoking^c^	93 (50.8）	4 (2.0）	0.000	104 (47.5）		2 (1.1）	0.000
Alcohol^c^	72 (39.3）	10 (5.0）	0.000	82 (37.4）		4 (2.3）	0.000
History of hypertension^c^	56 (30.6）	40 (19.8）	0.015	115 (52.5）		91 (51.4）	0.828
History of diabetes^c^	14 (7.7）	17 (8.4）	0.783	33 (15.1）		48 (27.1）	0.003
History of cardiac surgery^c^	9 (4.9）	13 (6.4）	0.522	18 (8.2）		20 (11.3）	0.301
History of myocardial infarction^c^	12 (6.6)	2 (1.0)	0.004	28 (12.8）		8 (4.5）	0.004
Preoperative hemoglobin (g/L)^a^	140.6 ± 17.8	125.0 ± 16.1	0.000	132.3 ± 17.4		122.5 ± 16.0	0.000
Preoperative sCr (umol/L)^a^	80.0 ± 14.7	61.1 ± 11.7	0.000	81.8 ± 14.3		66.2 ± 14.5	0.000
eGFR^a^	96.9 ± 15.7	100.2 ± 16.0	0.043	82.5 ± 12.1		80.8 ± 14.6	0.204
Preoperative LVEF（%）^b^	64.6 (58.0,68.6)	64.7 (60.6,68.0)	0.392	61.4 (55.1,66.4)		64.0 (57.9,67.4)	0.011
Operation type			0.032				0.001
VHD^c^	108 (59.0)	120 (59.4)		110 (50.2)		104 (58.8)	
CABG^c^	12 (6.6)	5 (2.5)		60 (27.4)		24 (13.6)	
CHD^c^	18 (9.8)	36 (17.8)		0		5 (2.8)	
Combined^c^	45 (24.6)	41 (20.3)		49 (22.4)		44 (24.9)	
**Intraoperative related characteristics**							
CPB duration (min)^b^	155 (115,201)	145 (115,181)	0.040	166 (133,208)		159 (118,205)	0.087
Lowest intraoperative hemoglobin (g/L)^a^	9.1 ± 1.2	7.6 ± 1.0	0.000	8.3 ± 1.1		7.4 ± 0.9	0.000
Intraoperative maximum lactic acid (mmol/L)^a^	1.8 ± 1.0	1.8 ± 0.9	0.845	1.7 ± 0.9		2.0 ± 1.3	0.029
Concentrated packed red blood cells^c^	10 (5.5）	59 (29.2）	0.000	42 (19.2）		87 (49.2）	0.000
Plasma transfusion^c^	50 (27.3）	49 (24.3）	0.492	107 (48.9）		78 (44.1）	0.342
Repeated CPB^c^	8 (4.4）	6 (3.0）	0.463	10 (4.6）		11 (6.2）	0.467

Abbreviations: AKI acute kidney injury, BMI body mass index, AKI acute kidney injury, sCr serum creatinine, LVEF left ventricular ejection fractions, GFR glomerular filtration rate, VHD valvular heart disease, CABG coronary artery bypass grafting, CHD congenital heart disease, CPB cardiopulmonary bypass.

a Values given as means ± standard deviation.

b Values given as median (25–75th percentile).

c Values given as count (percentage).

During the procedure, certain intraoperative characteristics were observed. Young males had a longer duration of cardiopulmonary bypass (CPB) compared to young females. Females in both age groups exhibited lower levels of intraoperative hemoglobin and a higher incidence of concentrated packed red blood cell transfusion. Additionally, old females had higher levels of intraoperative lactic acid compared to old males. (Table 1).

The study found that prior to Propensity Score Matching, 48 (26.23 %) male patients in the young group experienced AKI after surgery, compared to 27 (13.37 %) female patients, with a statistically significant difference. The relative risk (RR) for male patients was 1.962 [95 % CI 1.280, 3.008], P = 0.001.

In the old group, 95 (43.38 %) male patients and 66 (37.29 %) female patients developed AKI after surgery, with no significant difference observed (RR: 1.163 [95 % CI 0.912, 1.484], P = 0.220). Female patients in both age groups showed significantly lower SCr concentrations 15 days after surgery. In the older age group, the duration of postoperative endotracheal intubation was longer in females. (Table 2).

**Table 2:** Poor prognosis before Propensity Scroe Mathing.

Characteristics	Young group (age<60)				Old group (age≥60)		
	Male	Female	*P* value	Male		Female	*P* value
	(n = 183）	(n = 202）		(n = 219)		(n = 177）	
**Postoperative characteristics**							
Cerebral infarction^c^	2 (1.1）	3 (1.5）	0.911	6 (2.7）		1 (0.6）	0.212
Bleeding^c^	2（1.1）	4（2.0）	0.772	1 (0.5）		4 (2.3）	0.252
Postoperative AKI							
All states^c^	48 (26.2）	27 (13.4）	0.002	95 (43.4）		66 (37.3）	0.220
Stage 1^c^	39 (21.3）	16 (7.9）	0.117	78 (35.6）		49 (27.7）	0.478
Stage 2^c^	6 (3.3）	7 (3.5）		14 (6.4）		13 (7.3）	
Stage 3^c^	3 (1.6）	4 (2.0）		3 (1.4）		4 (2.3）	
15 days after operation sCr (umol/L)^a^	80.2 ± 25.3	60.9 ± 18.7	0.000	84.2 ± 22.5		70.1 ± 29.5	0.000
CRRT^c^	1 (0.5)	0	0.475	2 (0.9)		2 (1.1)	1.000
Hospital stay (days)^b^	21 (16,25）	19 (15,25）	0.188	23 (19,29）		23 (19,28）	0.833
ICU stay (days)^b^	4 (3,5）	4 (3,5）	0.729	5 (4,6）		5 (4,6）	0.288
Endotracheal intubation duration (hours)^b^	18 (13,23）	18 (15,22）	0.779	19 (16,29）		21 (18,37）	0.025
In-hospital mortality^c^	2 (1.1)	0	0.225	1 (0.5)		1 (0.6)	1.000

Abbreviations: CRRT continuous renal replacement therapy, ICU intensive care unit.

a Values given as means ± standard deviation.

b Values given as median (25–75th percentile).

c Values given as count (percentage).

### 3.3 Comparison of gender differences after propensity score matching

After adjusting for propensity scores, there was a considerable improvement in covariate balance (P > 0.1), indicating a significant enhancement in the covariate balance. Our results show no significant difference in AKI incidence between young males (24.1 %) and females (19.3 %) (RR: 1.249, [95 % CI, 0.698–2.239], P = 0.451). However, there is a significant difference in AKI stage between young males and females, with young women experiencing a higher stage of AKI than young men. In the old group, there was a similar incidence of AKI between males (43.4 %) and females (42.2 %) (RR: 1.028, [95 % CI, 0.723–1.463], P = 0.875). Additionally, female patients in both age groups had significantly lower SCr concentrations 15 days after surgery. Finally, in the old group, females had a longer duration of postoperative endotracheal intubation. (Table 3).

**Table 3:** Demography, clinical characteristics, and poor prognosis after trend matching.

Characteristics	Young group (age<60)			Old group (age≥60)		
	Male (n = 83)	Female (n = 83)	*P* value	Male (n = 83)	Female (n = 83)	*P* value
Patient-related characteristics						
Age（years）^b^	49 (39,56）	49 (42,55）	0.708	67 (63,71）	68 (65,71）	0.353
BMI (kg/m^2^)^a^	23.0 ± 3.2	23.5 ± 3.2	0.929	22.9 ± 2.7	23.1 ± 3.5	0.701
Smoking^c^	4 (4.8）	4 (4.8）	1.000	3 (3.6）	2 (2.4）	0.650
Alcohol^c^	14 (16.9）	8 (9.6）	0.170	5 (6.0）	4 (4.8）	0.732
History of hypertension^c^	13 (15.7)	14 (16.9)	0.833	41 (49.4)	45 (54.2)	0.534
History of diabetes^c^	8 (9.6)	5 (6.0)	0.386	12 (14.5)	14 (16.9)	0.669
History of cardiac surgery^c^	4（4.8）	4（4.8）	1.000	11 (13.3）	10 (12.0）	0.815
History of myocardial infarction^c^	2（2.4）	1（1.2）	0.560	6 (7.2）	5（6.0）	0.755
Preoperative LVEF（%）^b^	65.0 (60.1,68.8)	64.0 (60.3,68.0)	0.498	62.1 (56.0,67.8)	61.7 (56.7,66.4)	0.501
eGFR^a^	98.7 ± 16.4	101.4 ± 16.5	0.294	81.5 ± 12.4	82.5 ± 13.1	0.629
Operation type						
VHD^c^	50 (60.2)	52 (62.7)	0.985	57 (68.7)	50 (60.2)	0.335
CABG^c^	2 (2.4)	2 (2.4)		11 (13.3)	10 (12.0)	
CHD^c^	12 (14.5)	12 (15.5)		0	0	
Combined^c^	19 (22.9)	17 (20.5)		15 (18.1)	23 (27.7)	
CPB duration (min)^b^	146 (112,189)	148 (121,180)	0.837	146 (124,200)	159 (119,205)	0.672
Concentrated packed red blood cells^c^	5 (6.0）	6 (7.2）	0.755	11 (13.3）	18 (21.7）	0.152
Plasma transfusion^c^	18 (21.7）	19 (22.9）	0.852	37 (44.6）	38 (45.8）	0.876
Repeated CPB^c^	4 (4.8）	3 (3.6）	0.699	2 (2.4）	3 (3.6）	0.650
Postoperative characteristics						
Postoperative AKI						
All states^c^	20 (24.1）	16 (19.3）	0.451	36 (43.4）	35 (42.2）	0.875
Stage 1^c^	17 (20.5）	6 (7.2）	0.010	31 (37.3）	25 (30.1）	0.317
Stage 2^c^	1 (1.2）	6 (7.2）		4 (4.8）	8 (9.6）	
Stage 3^c^	2 (2.4）	4 (4.8）		1 (1.2）	2 (2.4）	
15 days after operation sCr (umol/L)^a^	77.6 ± 23.9	63.8 ± 24.7	0.000	83.8 ± 21.0	69.6 ± 31.4	0.001
CRRT^c^	0	0	–	0	1 (1.2)	0.316
Cerebral infarction c	1 (1.2)	0	0.316	1 (1.2)	0	0.316
Bleeding^c^	2（2.4）	2（2.4）	1.000	1 (1.2）	4 (4.8）	0.173
Hospital stay (days)^b^	20 (16,24)	19 (16,26)	0.951	23 (18,29)	23 (19,29)	0.580
ICU stay (days)^b^	4 (3,5)	4 (3,5)	0.883	5 (4,6)	5 (4,6)	0.840
Time of endotracheal intubation (hours)^b^	17 (11,21）	17 (15,21）	0.240	19 (16,27）	21 (18,41）	0.049
In-hospital mortality^c^	0	0	–	0	1 (1.2)	0.316

a Values given as means ± standard deviation.

b Values given as median (25–75th percentile).

c Values given as count (percentage).

### 3.4 Comparison between aki and non-aki groups

To investigate potential factors that could impact the incidence of AKI, we compared the baseline characteristics of patients with and without AKI. Table 4 illustrates the preoperative patient characteristics, revealing that the AKI group was older and had a higher BMI. Additionally, this group exhibited a lower eGFR and preoperative hemoglobin levels. Moreover, we found that the proportion of male patients, smokers, hypertensive individuals, those with diabetes mellitus, and those who underwent cardiac surgery or experienced myocardial infarction were higher in the AKI group.

**Table 4:** Preoperative and intraoperative characteristics of patients with and without AKI.

Parameters	No AKI (n = 545)	AKI (n = 236)	X/t	*P* value
Age (years)^a^	55 ± 13.2	62.5 ± 11.3	−6.705	0.000
Sex (males) b	259 (47.5)	143 (60.6)	11.263	0.001
Height (cm)^a^	162.0 ± 8.3	162.6 ± 8.4	−0.830	0.407
Weight (kg)^a^	59.3 ± 10.5	61.5 ± 10.8	−2.625	0.009
BMI(kg/m^2^)^a^	22.5 ± 3.1	23.2 ± 3.4	−2.724	0.007
Smoking^b^	125 (22.9)	78 (75.4)	8.759	0.003
Alcohol^b^	109 (20.0)	59 (25.0)	2.439	0.118
History of hypertension^b^	179 (32.8)	123 (52.1)	25.798	0.000
History of diabetes^b^	64 (11.7)	48 (20.3)	9.906	0.002
History of cardiac surgery^b^	33 (6.1)	27 (11.4)	6.735	0.009
History of myocardial infarction^b^	26 (4.8)	24 (10.2)	8.011	0.005
Preoperative LVEF<40^b^	17 (3.1)	11 (4.7)	1.132	0.287
Pulmonary hypertension^b^	163 (29.9)	76 (32.2)	0.408	0.523
sCr (umol/L)^a^	71.3 ± 15.8	75.3 ± 17.6	−3.152	0.002
eGFR^a^	91.9 ± 16.8	86.0 ± 16.5	4.552	0.000
Preoperative hemoglobin(g/L)^a^	131.1 ± 17.8	128.0 ± 18.8	2.210	0.027
Preoperative Coronary angiography^b^	297 (54.5)	146 (61.9)	3.643	0.056
Preoperative Vancomycin^b^	112 (20.6)	58 (24.6)	1.567	0.211
Operation type				
VHD^b^	306 (56.1)	136 (57.6)	16.498	0.001
CABG^b^	60 (11.0)	41 (17.4)		
CHD^b^	53 (9.7)	6 (2.5)		
Combined^b^	126 (23.1)	53 (22.5)		
CPB duration (min)^a^	157.4 ± 56.7	180.3 ± 76.9	−4.636	0.000
Aortic block time(min)^a^	117.5 ± 51.5	134.9 ± 65.3	−3.982	0.000
CPB maximum lactic acid(mmol/L)^a^	1.7 ± 0.9	2.0 ± 1.3	−3.508	0.000
CPB minimum residual alkali(mmol/L)^a^	−2.4 ± 2.1	−2.8 ± 2.3	2.669	0.008
CPB minimum hemoglobin(g/L)^a^	8.2 ± 1.3	7.9 ± 1.2	3.017	0.003
Concentrated packed red blood cells^b^	122 (22.4)	76 (32.2)	8.388	0.004
Plasma transfusion^b^	181 (33.2)	103 (43.6)	7.747	0.005
Repeated CPB^b^	18 (3.3)	17 (7.2)	5.854	0.016

a Values given as means ± standard deviation.

b Values given as count (percentage).

Patients with AKI and those without AKI underwent different surgical types. Upon examining the intraoperative characteristics, we discovered that the AKI group had a longer duration of cardiopulmonary bypass (CPB), aortic block time, and higher levels of lactic acid. Additionally, the AKI group had lower residual alkali and hemoglobin levels. Furthermore, we observed that the proportion of patients who received concentrated packed red blood cells, plasma transfusions, and underwent repeated CPB were higher in the AKI group (Table 4).

### 3.5 Multivariate logistic regression analysis of factors related to AKI in the young and old groups

Perform logistic regression analysis on factors with p < 0.05 in the univariate analysis of preoperative and intraoperative baseline characteristics between AKI and non-AKI groups. The risk factors identified for postoperative AKI were age [odds ratio (OR) 1.035; 95 % confidence interval (CI) 1.014–1.055; P = 0.001], male gender [OR 2.187; 95 % CI 1.394–3.433; P = 0.001], BMI [OR 1.086; 95 % CI 1.027–1.149; P = 0.004], and intraoperative hemoglobin levels [OR 0.723; 95 % CI 0.593–0.880; P = 0.001] (Table 5).

**Table 5:** Multivariate Analysis of Risk Factors for the patients with cardiac surgery.

Variable	B value	Standard error of B value	P value	OR value	OR value 95%CI
Age	0.034	0.010	0.001	1.035	1.014–1.055
Sex,Male	0.783	0.230	0.001	2.187	1.394–3.433
BMI	0.083	0.029	0.004	1.086	1.027–1.149
Smoking	0.200	0.225	0.373	1.222	0.786–1.897
HBP	0.264	0.188	0.159	1.302	0.902–1.881
DM	0.357	0.239	0.136	1.429	0.894–2.282
History of cardiac surgery	0.461	0.302	0.127	1.586	0.877–2.867
History of myocardial infarction	0.247	0.333	0.458	1.280	0.667–2.456
eGFR	0.001	0.007	0.868	1.001	0.988–1.014
Operation type					
VHD	0.196	0.214	0.360	1.216	0.800–1.849
CABG	−0.259	0.300	0.388	0.771	0.428–1.390
CHD	−0.301	0.498	0.546	0.740	0.279–1.964
CPB duration	0.003	0.002	0.075	1.003	1.000–1.006
Intraoperative maxmum lactic acid	0.133	0.090	0.137	1.142	0.9958–1.362
Intraoperative minimum hemoglobin	−0.325	0.101	0.001	0.723	0.593–0.880
Concentrated packed red blood cells	0.041	0.259	0.873	1.042	0.628–1.731
Plasma transfusion	−0.127	0.197	0.518	0.880	0.598–1.296
Repeated CPB	0.206	0.412	0.617	1.229	0.548–2.754

## 4 Discussion

This retrospective observational study found that, prior to adjusting for confounding factors, there were differences in many aspects of lifestyle habits such as smoking and drinking, BMI, past chronic diseases such as hypertension and diabetes, history of previous cardiac surgery, type of current cardiac surgery, CPB duration, and baseline renal function between male and female patients. Before adjusting for confounding factors, the incidence of AKI was lower in young women than in young men, suggesting that young women may have better outcomes than men. However, older women had longer tracheal intubation times, and there was no observed gender difference in AKI incidence in the elderly population.

We used PSM analysis to reduce the impact of confounding variables on the results and to draw more reliable conclusions [17]. After PSM processing, we observed that the preoperative and intraoperative baseline characteristics between male and female patients in both groups were balanced. The difference in the incidence of AKI between male and female patients in both groups was not statistically significant after PSM processing. However, young female patients had a higher stage of AKI, and older female patients had longer tracheal intubation times after surgery. This suggests that female patients with AKI may have more severe disease compared to male patients, which is consistent with the findings of many related studies [18,19].

Subsequently, we conducted univariate analysis of the preoperative and intraoperative baseline characteristics between patients with and without AKI, and performed logistic regression analysis on factors with p < 0.05 in the univariate analysis. We found that age, male gender, and BMI were independent risk factors, while intraoperative hemoglobin was a protective factor. These results are consistent with the findings of studies on risk factors for postoperative AKI in cardiac surgery both domestically and internationall [[20], [21], [22], [23], [24], [25], [26], [27], [28]].

As women reach menopause at an average age of 46–52, hormonal changes continue for several years after their last menstrual period. Additionally, when they are over 60 years old, there is a rapid decline in sex hormones in their bodies, and the protective effect of estrogen on the kidneys is weakened [13,14]. Therefore, we divided the study population into a young group (less than 60 years old) and an elderly group (60 years old or older). We observed the effect of gender on adverse outcomes after cardiac surgery in both the young and elderly groups.

An increasing number of studies have reported that estrogen and estrogen receptors may have a protective effect on renal ischemia-reperfusion injury (IRI) through multiple mechanism [29]. Renal IRI is a brief period of renal ischemia followed by restoration of blood flow and oxygen delivery, which leads to a series of harmful cellular events such as ROS production, inflammatory cytokine release, and tubular cell death, resulting in AKI. It has been increasingly reported that estrogen and estrogen receptors may exert a protective effect on renal IRI through multiple mechanisms [30]. The main cause of AKI after cardiovascular surgery is renal ischemia-reperfusion injury. Gender differences in AKI caused by IRI may be related to endogenous estrogen suppressing the renal sympathetic nervous system and reducing local norepinephrine level [31]. Pre-treatment with estrogen before inducing IRI can provide renal protection in female mice and castrated male mice. Studies have also shown that estrogen administration after cardiac arrest and cardiopulmonary resuscitation can improve AKI in male and female mice. In contrast, testosterone increases the sensitivity of renal IRI by inhibiting the activation of nitric oxide synthase and the Akt signaling pathway [32].

A large number of animal experiments have shown that estrogen and estrogen receptors have a protective effect on the kidney [[33], [34], [35], [36], [37]]. Many epidemiological studies, however, have shown that women have a higher risk of mortality or adverse events after cardiac surgery [18]. The results are contradictory. Our study also yielded such results: young women had a higher AKI stage, and elderly women had longer tracheal intubation time after surgery, after excluding the effects of confounding factors between genders. The possible reason is that women in southern China have smaller height and weight, smaller blood volume, and serious blood dilution and anemia during CPB preloading and intraoperative cardioplegic solution perfusion. Kidney tissue, especially the renal medulla, is highly sensitive to ischemia and hypoxia, and severe blood dilution and low hemoglobin levels lead to reduced kidney oxygen supply [38,39]. We also found that higher intraoperative hemoglobin levels were protective against AKI. In addition, women are more likely to receive transfusions of concentrated red blood cells than men, and blood transfusions can cause an inflammatory response and damage to the kidneys.

Lower baseline serum creatinine and BMI in females can confound the incidence of AKI observed in females as compared to males. Firstly, lower baseline serum creatinine levels in females may underestimate the incidence of AKI compared to males [40]. Since AKI is defined based on an increase in serum creatinine from baseline, if females start with lower baseline levels, they may not meet the diagnostic criteria for AKI even if they experience a significant increase in serum creatinine. This could result in an underestimation of AKI incidence in females. Secondly, the lower BMI in females may affect the distribution and metabolism of medications, including nephrotoxic agents, which can impact the occurrence of AKI. Different drug dosing strategies based on body weight may lead to variations in drug exposure and potential nephrotoxic effects [41,42]. Therefore, the lower BMI in females may contribute to a different risk profile for AKI compared to males.

Our study is a single-center observational study, and the sample selection was not random. The geographical region covered by the samples is limited, which may not fully represent the overall population of the study subjects. The main limitation is the presence of confounding bias. Although propensity score matching can reduce selection bias, it is difficult to completely eliminate it. Additionally, after propensity score matching, the sample size is smaller than the original sample size by 50 %, which may artificially introduce selection bias [43,44]. Furthermore, elderly females have longer duration of endotracheal intubation and weaker creatinine generation capacity compared to males, which may result in an overestimate of the association between gender and AKI. Our study indeed has limitations, and in future research, the use of multicenter large-sample databases can help reduce selection bias.

As this study was retrospective, there was a lack of clinical data on postmenopausal women who had previously used estrogen replacement therapy and women who had undergone oophorectomy before menopause. Therefore, these patients were not excluded. Long-term follow-up of renal function after surgery in AKI patients was also not achieved. In future studies, we will conduct more in-depth research.

The cardiac function of patients and the use of certain positive inotropic agents can have an impact on renal perfusion and potentially influence the incidence of AKI [[45], [46], [47], [48]]. Due to certain limitations, we were unable to collect information on this aspect. If conditions permit in the future, we will conduct relevant research in this area.

## 5 Conclusion

We have come to the following conclusions: in the population under 60 years old after excluding confounding factors, there is no significant difference in the incidence of AKI after cardiac surgery between females and males, but the AKI staging is higher in females. However, in the population aged 60 and above, there is no significant difference in the incidence and staging of AKI between females and males, but the intubation time after surgery is longer in females. Age, male gender, and BMI are independent risk factors, while intraoperative hemoglobin level is a protective factor in the overall population.

## Data availability statement

The original data was registered, and posted on Figshare (URL: www.figshare.com; https://doi.org/10.6084/m9.figshare.24225280).

## Ethics statement

All procedures in this study were in accordance with the Helsinki Declaration (revised in 2013), and the Ethics Committee at the First Affiliated Hospital of Wenzhou Medical University approved the study. The ethics approval number is KY2023-R214. The full name of the ethics committee is Ethics Committee in Clinical Research of the First Affiliated Hospital of Wenzhou Medical University.

## Funding/Financial support

This work was supported by Zhejiang medical and health science and technology project （NO.2022LY891); Wenzhou science and Technology Bureau project (NO. Y20220216); Clinical research project of Wenzhou Society of Integrated Traditional and Western Medicine (NO.2021001).

## CRediT authorship contribution statement

**Yichuan Wang:** Writing – original draft. **Xuliang Huang:** Formal analysis, Data curation. **Shanshan Xia:** Writing – review & editing. **Qingqing Huang:** Writing – review & editing. **Jue Wang:** Writing – original draft, Formal analysis. **Maochao Ding:** Formal analysis. **Yunchang Mo:** Writing – original draft, Data curation. **Jianping Yang:** Writing – review & editing, Writing – original draft.

## Declaration of competing interest

The authors declare that they have no known competing financial interests or personal relationships that could have appeared to influence the work reported in this paper.

## Figures and Tables

**Figure:**